# Relationship Between MR Spectroscopy-Detected Glutamatergic Neurometabolites and Changes in Social Behaviors in a Pilot Open-Label Trial of Memantine for Adults With Autism Spectrum Disorder

**DOI:** 10.3389/fpsyt.2022.898006

**Published:** 2022-07-22

**Authors:** Neetu Nair, John Patrick Hegarty, Carmen Mihaela Cirstea, Meng Gu, Carrina Brooke Appling, David Quentin Beversdorf

**Affiliations:** ^1^Interdisciplinary Neuroscience Program, University of Missouri, Columbia, MO, United States; ^2^Department of Psychiatry, University of Missouri, Columbia, MO, United States; ^3^Department of Psychiatry, School of Medicine, Stanford University, Palo Alto, CA, United States; ^4^Department of Physical Medicine and Rehabilitation, University of Missouri, Columbia, MO, United States; ^5^William and Nancy Thompson Endowed Chair in Radiology, Departments of Radiology, Neurology and Psychological Sciences, University of Missouri, Columbia, MO, United States

**Keywords:** autism, glutamate, magnetic resonance spectroscopy - MRS, social outcomes, memantine

## Abstract

**Background:**

The neurobiology underlying ASD is largely unknown but altered neural excitability/inhibitory ratios have been reported. Memantine is an N-methyl-D-aspartate (NMDA) glutamatergic antagonist studied for the treatment of core ASD symptoms, with mixed results. We examined whether glutamatergic levels were associated with and predicted response to memantine in an exploratory pilot study.

**Methods:**

Ten adult participants with ASD underwent proton magnetic resonance spectroscopy (^1^H-MRS) imaging at baseline and behavioral assessments before and after 12-weeks of open-label memantine. Post-treatment scores on Clinical Global Impressions–Improvement (CGI-I) for social interaction were the primary outcome measure, and scores on the Social Responsiveness Scale (SRS) were included as a secondary outcome. LCModel was used to quantify the concentrations of Point RESolved Spectroscopy-detected glutamate+glutamine (Glx) (and other neurometabolites, i.e., N-acetylaspartate, NAA; creatine+phosphocreatine, Cr+PCr, and myo-inositol, Ins), within the left dorsolateral prefrontal cortex (LDLPFC) and right (R) posterolateral cerebellum. SPM was used to perform brain tissue segmentation within the spectroscopic voxels. CGI-I scores post-treatment were used to classify the participants into two groups, responders (scores 1–3; *n* = 5) and non-responders (scores 4–7, or withdrew due to increase behaviors; *n* = 5). Independent samples *t-*tests, partial correlations and linear hierarchical regression models (SPSS) were used to determine between-group differences in neurometabolite concentrations and associations between neurometabolites and behavioral scores.

**Results:**

Responders and non-responders did not significantly differ in Glx levels in any region of interest, but differed in NAA levels in LDLPFC (higher in responders vs. non-responders). Although changes in CGI-I social scores were not correlated with Glx in any region of interest, the linear hierarchical regression did reveal that Glx and Ins levels in LDLPFC were predictors of post-treatment CGI-I social scores. Changes in SRS scores were correlated with baseline Cr+PCr levels in the LDLPFC.

**Discussion:**

Our pilot data suggest that baseline Glx, a marker of glutamatergic neurotransmission, did not directly predict response to memantine for social outcomes in adults with ASD. However, interactions between Glx and the neurometabolite associated with glial integrity (Ins) may help predict treatment response. Further, those with highest baseline NAA, a putative neuronal marker, and Cr+pCr, a brain energy metabolism marker, were the best responders. These preliminary results may explain some of the mixed results reported in previous memantine trials in ASD. Future studies will need to examine these results in a larger sample.

## Introduction

Autism spectrum disorder (ASD) is a behaviorally defined, complex neurodevelopmental disorder characterized by early childhood onset of marked difficulties with social interaction and communication and the presentation of restrictive, repetitive patterns of behaviors and interests (1). Its phenotypic heterogeneity makes studying and treating ASD a challenging task (2). Currently, only two FDA approved drugs, risperidone and aripiprazole, are available to treat ASD and they are primarily used to manage severe irritability and aggression associated with ASD (3). Neither have shown conclusive benefit for the core features affecting social and communication skills in ASD and our understanding of the underlying neurobiology is only beginning to emerge. Some research indicates that a disrupted balance between excitation (glutamatergic) and inhibition (gamma amino-butyric acid, GABAergic) may be a primary underlying mechanism of the ASD phenotype in some individuals (4, 5). Further, postmortem studies of the ASD brain indicate potential alterations in these components of the excitation-inhibition balance in individuals with ASD (4, 6–8). For instance, a number of postmortem studies have indicated deficits in expression of glutamatergic markers, and altered minicolumnary morphometry specific to the dorsolateral prefrontal cortex of individuals with autism (9). Similarly, postmortem studies have also identified the cerebellum as a region where glutamate and GABAergic abnormalities are consistently identified in individuals with autism (6, 9). In fact, animal studies provide preliminary evidence that GABAergic abnormalities in the cerebellum are directly related to glutamate transmission and release in the prefrontal cortex in autism models indicating a possible interplay between these two important regions in the neuropathology of autism tied to the excitation-inhibition imbalance hypothesis (10).

^1^H-MRS is a non-invasive neuroimaging tool that can be used to examine biochemical profiles of brain tissue and has identified different neurometabolic alterations associated with different psychiatric and neurological conditions, including ASD (11). Specifically, 1H MRS studies in ASD have demonstrated alterations in various cortical and subcortical regions of the brain, including the left dorsolateral prefrontal cortex (12) and cerebellum (15) which have shown abnormal levels of the 1H-MRS-detected biomarkers of glutamatergic neurotransmission, Glx (glutamate-glutamine complex) or glutamate (11, 13–16). Thus, drugs that can modulate the balance between excitation and inhibition in the brain may be beneficial for some patients with ASD and the use of 1H-MRS to determine if levels of a glutamatergic biomarker in the cerebellum or dorsolateral prefrontal cortex can predict response to treatments that target the balance between excitation and inhibition in the brain would be an innovative breakthrough toward providing precision medicine treatments for patients with ASD.

Memantine, a moderate affinity N-methyl-D-aspartate (NMDA) glutamate receptor antagonist that attenuates glutamatergic excitation, is an FDA approved treatment for Alzheimer's disease and is known to improve communication abilities in this population (17). A few small studies have shown some effectiveness of memantine in the treatment of social and communication aspects of ASD (18–20). Considering these cognitive and behavioral outcomes and the impact of memantine on excitatory-inhibitory balance, there has been significant interest in the potential use of memantine to target core symptoms in patients with ASD. However, in a recent randomized, controlled trial, memantine was not effective for targeting social withdrawal in ASD (21). Nonetheless, it is possible that differences in treatment response could be due to the heterogeneity in the nature of the excitatory-inhibitory balance between different patients with ASD, thereby causing a variation in response. Perhaps our understanding of the discrepancies between the small studies that documented evidence of beneficial effects of memantine in treating social and communication deficits associated with ASD and the negative larger clinical trial could be clarified with better understanding of aspects of the biological heterogeneity of the disorder (18–21). In such a scenario, a biomarker to predict each individual patients' treatment response would be invaluable for increasing treatment efficacy and decreasing trial and error.

The current pilot, clinical follow-on study used ^1^H MRS to examine whether Glx was associated with and could be used to predict treatment response to memantine in an open-label trial in adults with ASD. Since there is robust evidence supporting the role for the dorsolateral prefrontal cortex (DLPFC), the cerebellum, and their interconnection in the excitation-inhibition balance neuropathology of autism as described previously (6, 9), these two regions were selected as the regions of interest (ROI) for the current study. Specifically, our previous work had shown that the ratio between excitation (Glx) and inhibition (GABA) in the right (R) cerebellar hemisphere as well as connectivity between the left (L) DLPFC and the R cerebellum were associated with measures of social communication, (32) suggesting that these areas are reasonable target regions of interest to examine whether MRS markers of glutamatergic neurotransmission might predict response on social communication to glutamatergic antagonists. Additionally, abnormalities in the posterolateral cerebellar hemispheres appear to be associated with language and social communication, (37, 38) and project to the contralateral DLPFC (39, 40), and both regions are implicated in ASD pathology (41). We hypothesized that Glx levels in the LDLPFC and the R cerebellum will be predictive of changes in scores on social assessments after 12 weeks of treatment with memantine in a sample of 10 adult participants with ASD. Specifically, we expected that those with the highest Glx in these regions of interest would be the best responders on the social domain for CGI-I. In addition to the primary hypotheses concerning Glx, we examined other neurometabolites assessing neuronal health, viability, and quantity - N-acetylaspartate (NAA) (34), glial integrity (higher levels reflecting astroglial activation, gliosis, and inflammation) - myo-inositol (Ins) (35), and brain energy metabolism (creatine is well-established for its role in energy metabolism, and phosphocreatine is a potent antioxidant) - creatine and phosphocreatine (Cr+PCr) (36), to explore how they might be involved in treatment response and whether models that incorporate multiple neurometabolites may account for more variation in treatment response (42).

## Methods

### Participants

Adolescent and adult patients with a confirmed diagnosis of ASD and who were willing to try memantine as an off-label clinical follow-on treatment were recruited through clinics at the Thompson Center for Autism and Neurodevelopmental Disorders, University of Missouri, Columbia, Missouri. General inclusion criteria were: (1) Age ≥ 16 years, (2) ASD diagnosis as per DSM V determined by clinician interview and confirmed with an Autism Diagnostic Interview-Revised (ADI-R) or Autism Diagnostic Observation Schedule (ADOS), and (3) Score <4 on the Clinical Global Impressions – Severity (CGI-S) scale indicating mild to moderate illness. The mild to moderate illness group was selected to ensure patient comfort and safety while taking the exploratory nature of the study into consideration. Exclusion criteria were: (1) Contraindications to MRI (Magnetic Resonance Imaging) (e.g., metallic implants, pacemakers, claustrophobia, pregnancy, lactation), (2) memantine intolerance or known hypersensitivity to memantine hydrochloride or to any components used in the formulation, and (3) medications that might interact with memantine. All procedures were approved by the University of Missouri Institutional Review Board and all participants (and legal guardians, for participants <18 years of age) provided written consent/assent, as applicable.

### Measures

At baseline, participants were assessed on the following social and behavioral measures:

*The Clinical Global Impressions – Severity (CGI-S)* is a clinician rated scale (range one to seven, with one being no symptoms and seven being the most severe symptoms possible) to assess severity of symptoms, such as, social interaction, sensory sensitivities, restricted interests, verbal and non-verbal communication, etc. and is commonly used in ASD research (19, 21, 22). The CGI-S for social behavior was the focus in this study.

*The Social Responsiveness Scale (SRS)* is a well-validated 65 item questionnaire that specifically evaluates social deficits associated with ASD. Several studies in ASD have used SRS to track social outcomes in response to pharmacological interventions (21–29).

### Imaging

**Figure 1:**
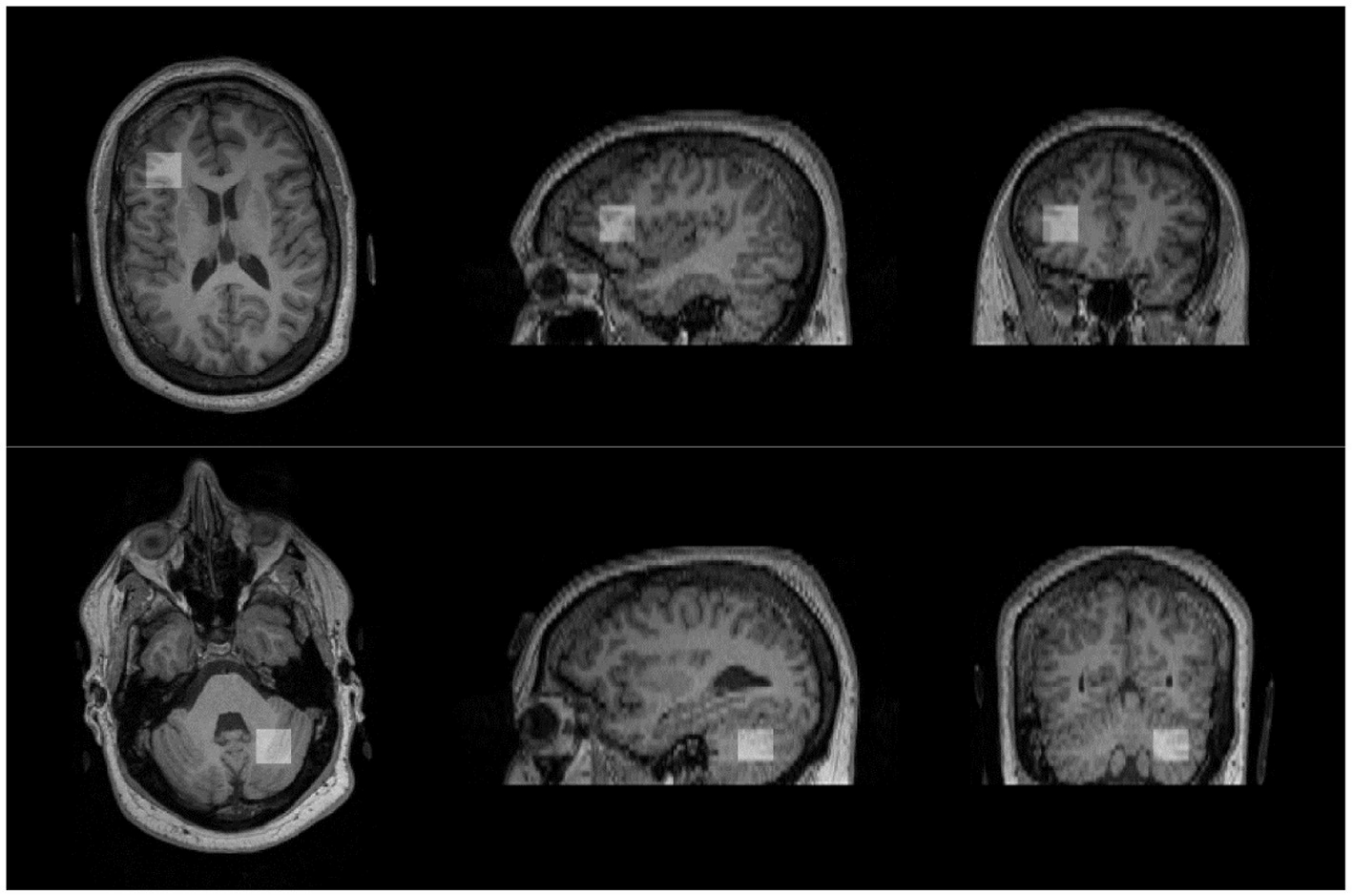
Approximate positions of voxels placed in the regions of interest. Voxel of interest (indicated by white square) placed in the left dorsolateral prefrontal cortex (top) and the right cerebellum (bottom).

Following baseline clinical assessments, subjects underwent an MRI scan, including structural MRI and ^1^H-MRS on a Siemens 3-Tesla TIM Trio MRI scanner located in the Brain Imaging Center at the University of Missouri. Participants were asked not to consume any forms of caffeine or alcohol 8 h before the scanning to eliminate effects from caffeine/alcohol on neurometabolites. High-resolution T1-weighted structural images were acquired using the three-dimensional multiplanar rapidly acquired gradient echo (MP-RAGE) pulse sequence: repetition time (TR), 2,500 ms; echo time (TE), 438 ms; flip angle, 8°, 256 × 256 voxel matrix; field of view (FOV), 256 mm; 176 axial slices; thickness, 1 mm. These images were used to quantify the brain tissue composition within the spectroscopic voxel and exclude any pathology. Based on anatomical landmarks, single voxel spectroscopy (SVS, 2 × 2 × 2 cm^3^) with Point RESolved Spectroscopy (PRESS, TE = 80 ms, TR = 2,000 ms, 128 averages, flip angle=90°, water suppression bandwidth = 50 Hz, delta frequency = −2.3 ppm, bandwidth = 1,200 Hz) was prescribed to the right posterolateral hemisphere of the L cerebellum targeting crus I/11 and the LDLPFC based on frontal gyral markers (Figure 1), brain regions previously identified as revealing changes in Glx/GABA and connectivity associated with performance on social communication (32). The same trained research personnel (NN) positioned the voxels on all the participants during the scanning sessions. Levels of Glx and other metabolites (NAA, Ins, and Cr+PCr) were examined. To avoid lipid artifact from the skull, six outer voxel suppression saturation bands were applied around the SVS. Automated, followed by manual, shimming was performed to achieve an optimal full width at half maximum of <20 Hz of the water signal from the entire excitation volume. Internal reference water signal was also acquired by using non–water suppressed MRS imaging to calculate absolute concentrations of neurometabolites of interest.

### Memantine Administration

Following the baseline imaging and behavioral assessment, participants were administered memantine, starting at 5 mg/day doses, and titrated up over 28 days to 20 mg/day based on response and tolerability, for a period of 12 weeks.

### Follow-Up Assessments

Upon completion of the 12 weeks of memantine, the participants repeated clinical assessments using the CGI-S for social interaction and SRS. The CGI-I for social interaction (ratings from 1 to 7, with 4 being no change, and decreasing scores representing minimal (3), marked (2) and dramatic (1) improvement, and increasing scores similarly representing worsening) (19, 21, 22) was also assessed at this time point and the social interaction subscale of the CGI-I served as the primary outcome measure.

### Analyses

Absolute concentrations of each metabolite– Glx, NAA, Ins, and Cr+PCr – measured in our regions of interest (ROI): LDLPFC and R cerebellum were quantified using Linear Combination of Model Spectra (LCModel) software [V6.3 (30)] with a standard PRESS basis set and water as internal concentration reference (31). The metabolites quantified in this manner serve as an estimate of their concentrations within the examined ROI (30, 31). Each metabolite of interest was expressed in institutional units (IU; ~ millimoles per kilogram wet weight) for each ROI. Gray matter volume within spectroscopy voxel was quantified using SPM (Mathworks Inc.) and controlled for during the statistical analysis. Processing of spectroscopic data is described in detail in our previous work (32).

For this pilot study, we summarized variables (primary: Glx; secondary: NAA, Ins, Cr+PCr) and outcomes (primary: scores on CGI-I for social interaction; secondary: SRS) measures by mean and standard deviation. To address our main hypothesis, responders and nonresponders were compared for Glx (and other neurometabolites) concentrations in each ROI using independent samples *t*-test. Pearson correlation analysis was also used to examine the relationships between Glx in each ROI and (1) scores on the social interaction subscale of the CGI-I social post-treatment and (2) changes in scores on the SRS baseline vs. post-treatment. Stepwise linear hierarchical regression models were used to examine whether baseline concentrations of neurometabolites (Glx, NAA, Ins, and Cr+PCr) in the LDLPFC and the R cerebellum predicted changes in scores on outcome measures using SPSS (IBM Corp, v26).

## Results

**Figure 2:**
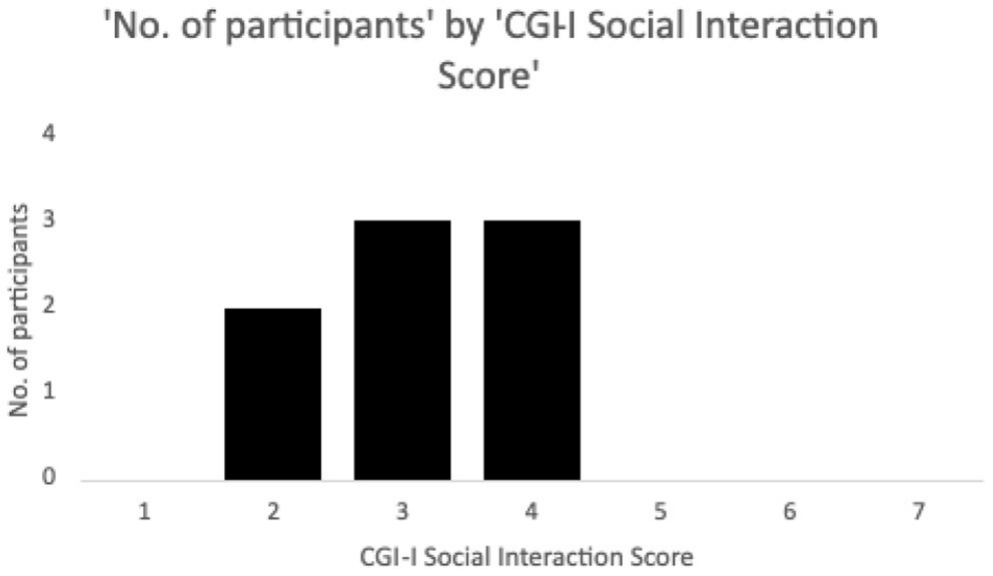
Number of participants by Clinical Global Impressions – Improvement (CGI-I) social interaction scores following 12 weeks of memantine treatment. Ratings on the scale range from 1 to 7, with 4 being no change, and decreasing scores representing minimal (3), marked (2) and dramatic (1) improvement, and increasing scores similarly representing worsening. Patients with scores 1–3 on the social interaction subscale were classified as responders while scores of 4–7 were considered non-responders. Two additional participants who discontinued the drug due to increased behaviors and did not complete the CGI-I were classed as non-responders but not shown in the Figure.

Ten participants (mean ± SD age = 24 ± 4 years, range 17–32 years old, one female, all Caucasian) were recruited to be a part of the study through the Thompson Center for Autism and Neurodevelopmental Disorders, University of Missouri, Columbia, Missouri. CGI-I scores on the social interaction subscale post 12 weeks of treatment with memantine were used to classify the participants into responders (scores 1–3, *n* = 5) and non-responders (scores 4–7, *n* = 3) (Figure 2).

Two subjects dropped out of the study at week 2 on memantine due to worsening behavioral symptoms, and were included in the non-responders group, as a result, for a total of *n* = 5 non-responders. No other side effects were reported.

All neurometabolites included in the analyses were within the limits required for spectra to be of acceptable quality: %SD <25 (%SD or Cramer Rao lower bounds, representing the threshold of the error associated with model fitting) and signal to noise >10. Due to head motion-related artifacts data from one participant had to be dropped from further analysis. Three other participants had high lipid contamination in the spectra acquired from the LDLPFC, suggesting tissue other than brain was included, and were not included in the corresponding models.

Overall, there were *n* = 9 participants with pre- and *n* = 7 participants with post-trial outcome data, of which *n* = 6 had high quality MRS data from the LDLPFC and *n* = 9 for the R cerebellum.

### Comparison of Responders and Non-responders

**Figure 3:**
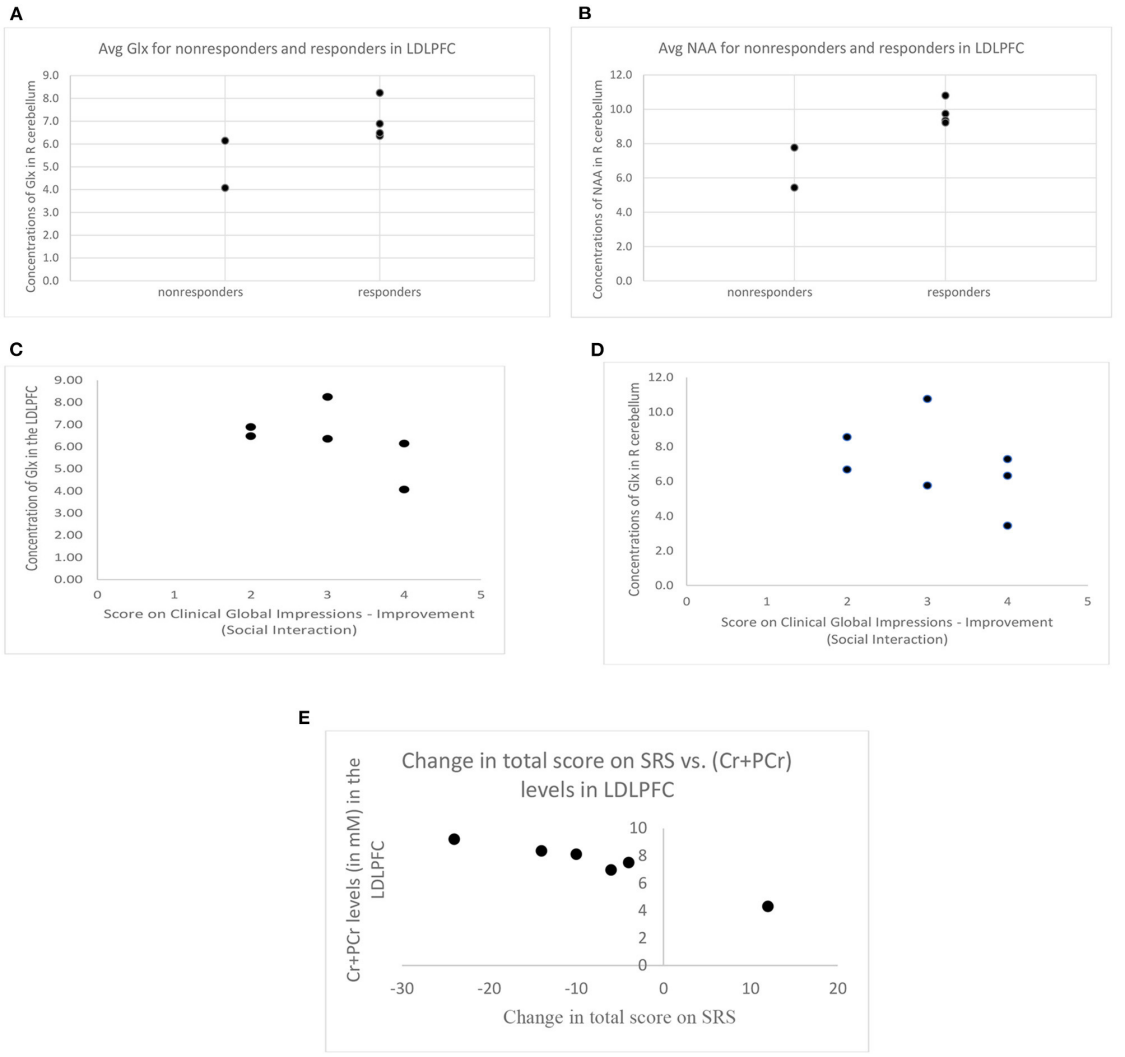
**(A)** Difference in Glx concentration between responders (CGI-I score of 3 or less) and non-responders (CGI-I score of 4 or more, or withdrew due to worsening behaviors) and **(B)** difference in NAA concentration between responders and non-responders (error bars represent standard deviation, * = *p* < 0.05, #= *p* ≤ 0.1). **(C)** Concentration of Glx in the LDLPFC and **(D)** concentration of Glx in the R cerebellum across CGI-I scores for all participants that do have follow-up visits allowing the obtaining of CGI-I scores. **(E)** Change in SRS total score (negative values indicate improvement posttreatment) was negatively associated with (Cr+PCr) levels in the LDLPFC. SRS, Social Responsiveness Scale. LDLPFC, Left dorsolateral prefrontal cortex. Glx, Glutamate and its precursor glutamine; LDLPFC, Left dorsolateral prefrontal cortex; R cerebellum, right cerebellum; Cr+PCr, Creatine + Phosphocreatine; SRS, Social Responsiveness Scale; CGI-I, Clinical Global Impressions-Improvement.

When responders and non-responders were compared, with the two participants dropping out due to worsening symptoms categorized as non-responders, no significant differences were observed for either of the two defined ROIs for Glx (LDLPFC: responders 7.00 ± 0.87 IU, non-responders 5.11 ± 1.47 IU, *t* = −2.08, *p* = 0.10 (see Figure 3A); R cerebellum: responders: 7.95 ± 2.21 IU, non-responders: 6.86 ± 2.86 IU, *t* = −0.60, *p* = 0.57). However, the number of participants with high quality data in the non-responder group was very limited for the LDLPFC, but if the *p*-value is to be believed, a larger sample size may reach statistical significance.

Our series of analysis of the secondary neurometabolites showed significantly higher levels of NAA in the LDLPFC in responders compared to the non-responders (9.78 ± 0.71 IU vs. 6.61 ± 1.65 IU, *t* = −3.56, *p* = 0.024) (see Figure 3B), again with a very limited number of non-responders with high quality data in the LDLPFC. A similar pattern, increased levels in responders vs. non-responders, was also observed for Cr+PCr levels in the R cerebellum; however, this difference did not reach statistical significance (responders: 12.34 ± 1.01 IU, non-responders 10.30 ± 1.35 IU, *t* = −2.13; *p* = 0.10).

### Correlations

Partial correlations controlling for age and gray matter volume fraction within ROI revealed that CGI-I scores on the social interaction subscale posttreatment were not directly correlated with Glx (LDLPFC: *r* = −0.76, *p* = 0.24 (see Figure 3C); R cerebellum: *r* = −0.45; *p* = 0.55 (see Figure 3D) or other neurometabolite levels at baseline in either of the two ROIs (*p* > 0.1 for all). The change in SRS score (calculated based on subscale T-scores, baseline vs. posttreatment) was negatively associated with baseline Cr+PCr levels in the LDLPFC (*r* = −0.956, *p* = 0.04) (see Figure 3E).

### Hierarchical Regression Models

Linear hierarchical regression models revealed that a final model with Glx (*B* = −1.07, *p* = 0.02) and Ins (*B* = 0.58, *p* = 0.04) in the LDLPFC at baseline significantly predicted scores on the social interaction CGI-I posttreatment (*R*^2^ adjusted = 0.86, *F* = 9.188, *p* = 0.05).

## Discussion

This pilot study examined whether ^1^H-MRS-detected Glx (and other neurometabolites) in two targeted brain regions was associated with or could predict treatment response to a moderate affinity NMDA receptor antagonist, memantine, in adults and adolescents with ASD. Five participants did respond (scores of 1–3 on the CGI-I for social interaction) while five did not respond (scores of 4–7, or stopped due to increased behaviors) to memantine. Our preliminary data did not reveal a relationship between baseline Glx levels in either ROI and response to memantine. However, Glx along with Ins within the LDLPFC was found to predict the post-treatment scores on the social interaction subscale of the CGI-I. Specifically, it appears that higher levels of Glx and lower levels of Ins in the LDLPFC were predictive of lower scores on the CGI-I (or greater improvement) following treatment with memantine.

Additionally, higher levels of Cr+PCr in the LDLPFC at baseline were associated with decreases in SRS total score post treatment. Higher NAA levels were also found in the LDLPFC in responders than in non-responders. Since creatine levels reflect cellular energy metabolism and NAA levels are connected to energy metabolism in neuronal mitochondria, these results may indicate that the effectiveness of memantine treatment was dependent on the level of mitochondrial dysfunction, a phenomenon commonly noted in ASD pathology (33), in the LDLPFC. However, these results will need confirmation in larger samples. Additionally, larger samples will allow the possibility of understanding how factors such as head circumference and intellectual functioning might relate to these findings.

These preliminary results are an initial effort to understand the mixed results reported in previous studies using memantine in ASD. It is possible that there exist different subsets of autism that respond differently to this treatment. Our hypothesis was that glutamatergic levels would predict response. However, the preliminary evidence suggests that there may be a more complex relationship, where increased glutamatergic levels, in the additional setting of altered glial and cellular energy metabolism markers, in the LDLPFC may show more improvements with memantine. Additionally, when responders were compared to non-responders (allowing inclusion of participants that withdrew due to not tolerating the medication), NAA levels in the LDLPFC differed between groups, providing further support that brain energy metabolism and neuronal integrity are important in predicting therapy response. Overall, these preliminary results raise the possibility of using 1H-MRS as a tool to discover potential biomarkers for treatment response in ASD. However, larger sample sizes will be needed to confirm these findings, and additionally to determine whether significant effects might be revealed for the relationship between Glx (and other neurometabolites) and treatment response with a more robust sample. For instance, a weak trend was observed for greater LDLPFC Glx among the good responders (*p* = 0.1). Based on these data, if the results from such a small sample are to hold true in further study, a sample size of 11 per group (responders, non-responders) would be sufficient for a power of 0.80 to yield a significant group difference in LDLPFC Glx at α = 0.05. Additionally, other neurometabolites, e.g., GABA, may be relevant, given the recent work demonstrating relationships between functional connectivity an excitatory/inhibitory balance in ASD (5, 32). While the sample size is small for extensive interpretation of these findings, future studies can explore whether response in the social domain to memantine might be related to glial function, as may be suggested by the relationship with Ins, whereby response is greatest with increased glutamate specifically in the setting of less activated microglia. The direction of this outcome is unexpected, as we would have predicted that patients with more activated microglia would have an augmented response to the inhibition of excitatory activity with an NMDA antagonist in the setting of increased baseline glutamate, do to putative compounding of the hyperexcitable state, so this further highlights the need for confirmation in future studies.

This line of work could, in turn, have important implications for clinical care including improving accuracy of individual prognosis and individualizing treatments in ASD. Recent studies have suggested that memantine might better target cognitive outcomes in ASD rather than social (18). However, the findings from the current study begin to raise the question as to whether social outcomes might still be relevant in an optimally targeted subset of patients.

The small sample size (particularly for 1H-MRS analysis) is a definite limitation of the current exploratory pilot study. This impacts the generalizability of our findings. Additionally, selection of patients capable of participating in the imaging session without sedation likely also introduces a bias in the findings, further impacting generalizability. Future studies should, therefore, be performed with larger samples. Additionally, while our study specifically targeted Glx since the drug memantine targets glutamate receptors, it would be critical for future work to gain a better understanding of the more complete role of the balance in excitation/inhibition in predicting the effects of memantine or related agents, including targeting GABA. Expanding the regions of interest to include other regions within the networks involved in social communication would also be critical in future studies, in addition to incorporating newer automated techniques for ROI optimization to improve outcomes.

## Data Availability Statement

The raw data supporting the conclusions of this article will be made available by the authors, without undue reservation.

## Ethics Statement

The studies involving human participants were reviewed and approved by University of Missouri. Written informed consent to participate in this study was provided by the participants' legal guardian/next of kin.

## Author Contributions

DB, JH, and NN designed the project. NN implemented the work with DB. CC oversaw the MRS aspects throughout. MG also helped guide the MRS analysis. CA helped to retrieve and prepare the data from the database in preparation for the analysis by MG. All authors contributed to the article and approved the submitted version.

## Funding

Research was supported by the University of Missouri Research Board Grant (PI: DB). ClinicalTrials.gov number: NCT02811627.

## Conflict of Interest

DB has received support for consulting with Impel Pharma, Stalicla Biosciences, Quadrant Biosciences, Scioto Pharma, and YAMO Pharma in the past year unrelated to this work. The remaining authors declare that the research was conducted in the absence of any commercial or financial relationships that could be construed as a potential conflict of interest.

## Publisher's Note

All claims expressed in this article are solely those of the authors and do not necessarily represent those of their affiliated organizations, or those of the publisher, the editors and the reviewers. Any product that may be evaluated in this article, or claim that may be made by its manufacturer, is not guaranteed or endorsed by the publisher.
